# Modeling the process of human tumorigenesis

**DOI:** 10.1038/ncomms15422

**Published:** 2017-05-25

**Authors:** Sneha Balani, Long V. Nguyen, Connie J. Eaves

**Affiliations:** 1 Terry Fox Laboratory, British Columbia Cancer Agency, Vancouver, British Columbia, Canada

## Abstract

Modelling the genesis of human cancers is at a scientific turning point. Starting from primary sources of normal human cells, it is now possible to reproducibly generate several types of malignant cell populations. Powerful methods for clonally tracking and manipulating their appearance and progression in serially transplanted immunodeficient mice are also in place. These developments circumvent historic drawbacks inherent in analyses of cancers produced in model organisms, established human malignant cell lines, or highly heterogeneous patient samples. In this review, we survey the advantages, contributions and limitations of current *de novo* human tumorigenesis strategies and note several exciting prospects on the horizon.

## Abstract (web summary)

A better understanding of the earliest stages of human cancer formation can enable future improvements in early detection, diagnosis and treatment. In this review, the authors summarize the methods enabling *de novo* tumorigenesis protocols to be applied to human cells and the insights derived from them to date, as well as the exciting and relevant technical developments anticipated to extend even further the utility of these strategies.

Identifying established malignancies in humans is usually not a difficult clinical issue as most human cancers do not become symptomatic until they are well advanced. Indeed, by the time they are detected, most will already consist of millions, if not trillions, of cells exhibiting many abnormal features^1^. These include evidence of invasive behaviour, deregulated growth, cells with an abnormal morphology and disorganized histology, and possession of a diversity of mutations^2^. How these can be usefully combined to generate more refined assessments of categories and stages of human cancer development has, however, challenged pathologists for decades. In addition, the molecular events involved in the early genesis of malignant human cell populations have been particularly elusive. This is because these stages are not usually detected in patients and, when they are, very little if any tissue is available for research studies. At the same time, there is expanding interest in the possibility that a better understanding of the initial changes that lead to an irreversibly transformed state and new ways to identify such changes might revolutionize early detection strategies as well as therapeutic success rates. Many approaches, both prospective and retrospective, have thus been developed to recreate and interrogate the process of tumorigenesis. All have particular advantages, but also significant caveats and shortcomings. What is new, are recent technological advances that are now enabling malignant populations of human cells to be generated *de novo* from primary tissue sources.

Here, we first review the background of information on which current understanding of the process of human oncogenesis has been founded. This is then followed by a review of newer developments and findings emanating from *de novo* tumorigenesis experiments that are driving new concepts relevant to this rapidly evolving topic. When coupled with unbiased DNA barcoding, reprogramming, and CRISPR/Cas9 technologies, these methods hold promise for obtaining further insights into the different stages of development of malignant human cell populations with unprecedented precision and clinical relevance.

## The Past—origin of current concepts

### Tumorigenesis viewed as an evolutionary process

**Figure 1:**
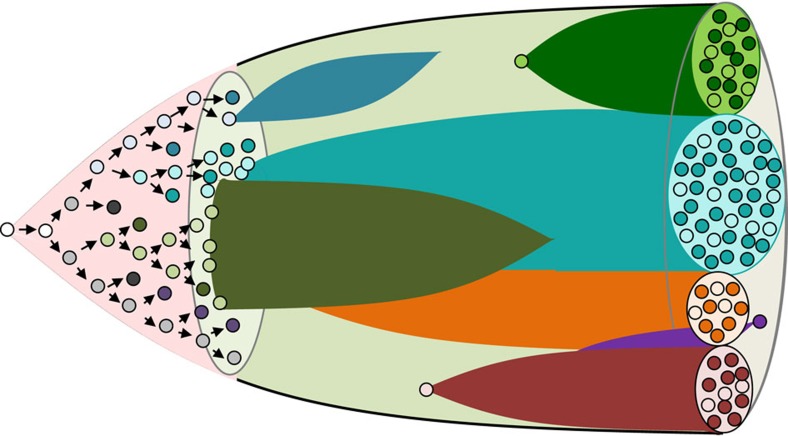
Schematic depiction of the subclonal evolution and diversification of cell types in developing malignant populations. In this diagram, subclones identified by accumulating genetic changes are shown by different colours. Cells within each clone that have proliferative potential are shown as pale cells in contrast to some of their progeny that can no longer divide that are shown as dark cells (to illustrate the diversification of biological properties that occurs both within and between subclones), with some clones being transient, whereas others are persistent but variably expanding.

The concept that almost all human tumours represent aberrant clonal outgrowths is well established^3^. However, this simply means that the malignant population that ultimately appears represents the deregulated growth of a single cell. It does not imply biologic or even genetic identity among its progeny. Nevertheless, it does make it likely that all members of the clone will carry a trace of the earliest genetic or epigenetic changes that drove its deregulated expansion. It is also important to remember that, by the time a cancer is first detectable, it will already contain many millions or even billions of cells produced through dozens of amplifying divisions. During this process, additional genetic diversification and evolution occurs (Fig. 1). This is due in part just to normal rates of incorrect DNA replication (estimated as 2.3 × 10^−8^ mutations per nucleotide per cell generation in human cells)^4^. A decreased control of DNA stability is also a common feature of malignant cells. Other mechanisms that contribute to the variable diversification of malignant clones include the tissue and genotype of the cell in which the process of transformation is initiated, the age of the individual in which this process begins and numerous environmental factors^5^,^6^,^7^,^8^,^9^.

Modern sequencing data has now revealed the enormous heterogeneity that exists within the genomes of malignant populations^10^. This heterogeneity is often apparent within a single cancer assessed at a single moment and sampled from a single site. Additional heterogeneity may also be encountered when different sites are examined, or the same tumour is sampled at different times, or from different individuals with tumours that have arisen in the same tissue. The genetic diversity that characterizes many malignant populations has multiple causes. One is that the full transformation of human cells relies on the accumulation of multiple mutations through a stepwise process in which different rare mutations and/or epigenetic changes must be accrued in order for cells to acquire a deregulated ability for unlimited division as well as other abnormal phenotypic properties^11^,^12^. From this concept, it has been inferred that the process of spontaneous oncogenesis in humans would most often be slow, at least initially, spanning a period of several years. Such a model, in turn, implies that most cancers would originate in a cell type that is normally long-lived but still has sufficient proliferative capacity to accumulate the number of rare mutations required to become transformed. Interestingly, it also implies the possible generation of neoplastic clones with mutations that predispose to further transformation but may never do so, as is being increasingly documented in many tissues^13^,^14^,^15^. However, accelerating the process, and a cause of more rapidly increasing genetic diversity, would be the acquisition of one or more mutations or epigenetic changes that compromise genomic stability^16^,^17^,^18^,^19^. At the same time, recent genomic analyses of some human cancers are suggesting alternative trajectories in which transforming populations early on may undergo cataclysmic bursts of genomic diversification^20^.

Most of our knowledge of the process of oncogenesis in human tissues has been derived historically from 3 different types of investigation: (i) genomic analyses of cells obtained from patients at different times during the development and evolution of their cancer with retrospective inferences drawn from the spectrum and prevalence of sequence changes (best determined at the single cell level)^21^,^22^,^23^,^24^; (ii) inferences derived from single cell or clonal transcriptome studies^25^,^26^; and (iii) studies of genetically engineered malignant populations in model organisms, and mice in particular^27^,^28^,^29^. The first two of these types of investigation have shown that most human cancers, by the time they become clinically evident, consist of cells that have already acquired multiple mutations^2^. However, the extent to which the time course or order in which the same mutations or other relevant (for example, epigenetically determined) changes are accrued in any given human cancer still remains poorly understood^30^.

Although, mutations do not occur randomly^31^,^32^,^33^, the likelihood of a given complement of mutations being accrued in the lifetime of an individual human cell or its progeny remains low. It is therefore not surprising that the more primitive cells in tissues of high cell turnover; that is, those cells responsible for sustaining the required cell outputs of the tissue, have commonly been assumed to serve as reservoirs of premalignant changes that accrue throughout life.

In the human hematopoietic system, this concept has been most strongly supported by genomic analyses of cells from patients with chronic myeloid leukemia (CML). This leukemia is unusual in that it is typically diagnosed at an early stage (referred to as chronic phase). Although the clone can already be huge (typically more than a trillion cells), the process of differentiation is minimally perturbed and hence most of the cells are actually highly differentiated, short-lived mature blood cells, indistinguishable from their normal counterparts. Cell production within the chronic phase clone, in fact, largely mirrors the normal process of adult human hematopoiesis and is likewise sustained by a phenotypically similar and rare subset of multi-potent self-renewing CML (*BCR-ABL1+*) cells as shown by their unique capacity for prolonged CML cell output both *in vitro* and *in vivo*^34^,^35^. Interestingly, effective treatment of CML patients with tyrosine kinase inhibitors (TKIs) selectively eliminates the bulk of the clone (>99.9%), but has less effect on the CML stem cells which can then rapidly regenerate an enlarged derivative population if TKI administration is transiently arrested^36^. Moreover, if the chronic phase clone is not adequately reduced, subclones of CML cells with additional mutations and biological properties eventually appear and produce an inevitably fatal acute lymphoid or myeloid leukemia^37^. Additional studies have suggested that at least some other forms of human acute myeloid leukemia (AML)^23^,^38^,^39^,^40^,^41^,^42^ myelodysplastic syndromes (MDS)^43^,^44^ fit a similar model of step-wise disease progression.

### The cancer stem cell concept

The restriction of transplantable tumour-initiating ability (in immunodeficient mice) to a rare, prospectively separable subset of malignant human cell populations was first demonstrated in human AML^45^. This was followed soon thereafter by analogous experimental evidence of tumour-initiating ability confined to distinct and prospectively separable subsets of cells in primary samples of human breast^46^, brain^47^ and colon cancers^48^. Together, these findings established the concept of cancer stem cells thus identified by their transplantable tumour-initiating property. Phenotypic and transcriptional similarities between the subsets of malignant human cells and the stem cells of the tissue of origin have reinforced the association of this ‘cancer stem cell' functionality with the retention or reactivation of molecular mechanisms essential to the maintenance of normal tissue stem cells. Of particular interest in this regard, has been an association of quiescence with many types of both normal and cancer stem cells^49^,^50^,^51^,^52^,^53^, thus accounting for the innate resistance of the cancer stem cell population to standard chemotherapeutic agents that primarily target dividing cells. The relative insensitivity of these cells to treatment also makes them dangerous reservoirs for the accumulation of additional mutations that make their elimination even more problematic.

However, in other human cancers, like melanoma^54^, a majority of the cells with self-sustaining tumorigenic properties is often already apparent at diagnosis. In addition, there is increasing evidence of the reversibility of the properties that confer transplantable tumour-initiating activity in a growing number of human tumours. This phenomenon, referred to as phenotypic instability or plasticity, is thought to reflect changes triggered by cues from the environment or stochastically regulated intrinsic parameters^55^,^56^,^57^,^58^, and may add significantly to the heterogeneous dynamic evolution of tumours caused by a stepwise and branched process of mutation accrual noted already.

### Transgenic mouse models of *de novo* tumorigenesis

Mouse models have been valuable for analysing early oncogenic events because of the ease with which their genomes can be genetically manipulated in defined manners and times. This has made it possible to interrogate many specific molecular, cellular and tissue-specific characteristics associated with inherited and somatically acquired mutations in humans that have been inferred to play a role in their transformation. There are, however, numerous physiological differences between mice and humans that limit the utility of this approach to provide insights into mechanisms operative in humans. Of particular note in this regard is the apparent ease with which mouse cells can be transformed, in contrast to their human counterparts^59^.

Mouse models have been particularly helpful in tracing the clonal origin of normal and transformed cells in various tissues. This type of experiment typically takes advantage of a known lineage-specific gene promoter to transgenically engineer the expression of an oncogene exclusively in the cell types in which that promoter is active. This strategy can thus be used to track subsequent changes in predefined cell types before the formation of a detectable tumour. In addition, this strategy has been useful for identifying the origin of any malignant populations ultimately obtained and demonstrating different properties in the tumours thus generated^27^,^60^,^61^,^62^,^63^.

For example, in the adult mammary gland, the specificity of expression of different types of cytokeratins associated with either basal or luminal mammary epithelial cells has enabled tumours derived from these different cell types to be produced and compared. These include the production of estrogen receptor-positive and progesterone receptor-positive (ER^+^PR^+^) and ER^−^PR^−^ tumours from mouse basal and luminal cells engineered separately to express *PIK3CA*(H1047R), thereby creating models that appear to mirror analogous phenotypes of human breast cancer^64^,^65^. However, it is also noteworthy that potent stimulation of normal adult mouse luminal mammary cells, even in the absence of a mutant gene, can induce their production of both basal and luminal progeny^66^, despite evidence of their more restricted differentiation behaviour *in vitro* and *in vivo*^67^,^68^. Similar transgenic models have been used to study concomitant *PIK3CA* expression and *p53* deletion^64^,^65^,^69^, as well as concomitant *BRCA1/PTEN/p53* depletion in mouse mammary cells^70^.

Familial adenomatous polyposis, a human condition with a recognized step-wise process of transformation, is another interesting example of a tumour that has been modelled in mice. In this case, the inducible loss of only one *APC* allele in Lrig1-expressing cells resulted first in their acquisition of pre-neoplastic changes and then their generation of multiple colonic tumours within 50 days^71^. In the epidermis, Sox2 expression has been found to initiate the *de novo* growth of a squamous cell carcinoma^72^, and glioblastoma in the brain^73^. On the other hand, forced expression of Sox9 in the epidermis was sufficient to induce the *de novo* formation of basal cell carcinomas^74^. Mouse models have been similarly used to study the ability of Wnt-activation^75^, oncogenic Ras^76^, Myb^77^, and Pgc-1b^78^ to initiate *de novo* tumorigenesis in intestinal epithelial cells. Studies in the hematopoietic system of mice have also reported analogous strategies for the *de novo* generation of AML^79^, plasma cell neoplasms^80^, and lymphoid malignancies^81^.

A key advantage of such transgenic mouse models of oncogenesis is their ability to mimic the effect of acquiring endogenous oncogenic mutations under homeostatic conditions. They also enable events critical to the initiation of tumour formation within the normal tissue architecture to be examined. However, it is also possible that the level of oncogene expression driven by promoters of lineage-specific (tracer) genes would be different from that characteristic of tumours caused by mutations in the corresponding endogenous genes. The different epigenetic mechanisms regulating their expression in the two different sites would be expected to contribute to the types, or at least speed, of the phenotypic changes induced. Furthermore, interactions of spontaneously arising mutant human cells within their native microenvironments, as well as intrinsic aspects of the process of normal tissue control, may not be well modelled in the mouse. On the other hand, transgenic mouse models offer the advantage that they can now be readily engineered to regulate not only the lineage, but also the level, timing and duration of expression of genes of interest that have been introduced into the germ line.

Mouse knock-in (KI) protocols now enable some of the short-comings of historic transgenic models to be addressed. These involve the use of homologous recombination to replace endogenous genes in mouse embryonic stem cells with candidate oncogenic mutant alleles and subsequent introduction of the selected cells into the mouse germ line. KI mice have thus been generated to explore the function of Kit mutations in gastrointestinal stromal tumours, the role of TP53 in Li-Fraumeni syndrome tumours and for recapitulating familial cancers. This technique has also been further adapted to induce spontaneous expression of the oncogenic *KRAS*(*G12D*) gene enabling the genesis of lung cancer, thymic lymphoma, and aberrant intestinal crypt foci to be created and studied^82^. CRISPR/Cas9 technology to introduce oncogenic mutations into the endogenous locus of the mouse germ line (with or without controlled expression) is now offering even greater precision in the types of genetic manipulations that can be deployed to analyse downstream biological effects^83^,^84^,^85^.

### Transformation of human cell lines

Much of the process of malignant transformation of human cells has historically focused on immortalized non-tumorigenic human cell lines as the starting material. MCF-10A is an example of a human mammary epithelial cell line of this type that has been used for this purpose. Forced over-expression of *MYC*^86^, *EGFR*^87^, *Nicastrin*^88^, *ERBB2* (ref. 89), and *RANK*^90^ in MCF-10A cells produced several phenotypic changes associated with transformation. These included changes in their growth properties in three-dimensional (3D) cultures^91^, as well as in xenotransplant models^89^. However, a study based on a non-tumorigenic human prostate cell line, EPT2-D5, showed that metastatic prostate tumours could be generated in xenografted mice simply by first culturing the cells in a protein-free media to generate ‘spheres'^92^. Moreover, recent epigenomic and transcriptional analysis of MCF-10A and two other immortalized (184-hTERT) non-tumorigenic human mammary cell lines has revealed that all three have acquired significant differences from primary sources of human mammary cells^93^. It thus remains unclear how well the transformation of such immortalized cell lines will reflect those relevant to the spontaneous genesis of cancers from primary sources of human cells.

## The present—utility of *de novo* human models

### External factors

It is also now clear that most malignant cells are not capable of autonomous growth, although the acquisition of autocrine properties and other intrinsic mechanisms for enhanced auto-stimulation and survival are common. Tumours, like normal tissues, also require a blood supply and frequently contain numerous other non-clonally derived cell types, even to the point where these latter cell types may become predominant^94^,^95^,^96^. These cells may include many different stromal cell types as well as the components of vessel walls and extravasated blood and lymphoid cells. External cues emanating from these different sources both positively and negatively regulate the local rate of growth of neighbouring malignant cells as well as their invasive behaviour^97^,^98^, thereby contributing to the overall dynamic evolution of the genomic, biologic and geographic properties of the entire population of malignant cells^10^.

### Persistence of tissue-specific programs

In addition to these multiple sources of cellular heterogeneity, malignant populations also frequently retain many of the distinguishing but heterogeneous features of the tissue from which they have arisen even after they have metastasized. In fact, almost by definition, a stepwise model of transformation would imply that this would have to be the case unless it completely froze the state of the first affected cell resulting in the generation of a clone of identical daughter phenotypes. If not, then it would be predicted that the composition of a malignant clone would also reflect some of the phenotypic heterogeneity determined by persisting elements of processes that normally sustain the maintenance, organization and differentiation of cells in that tissue^99^. Thus, the phenotypic heterogeneity inherent in many bulk malignant populations would be anticipated to also reflect some of the complex diversity of persisting epigenetic and transcriptional elements characteristic of the formation and maintenance of the tissue from which the cells arose. In this regard, it is noteworthy that different tissues make different utilization of multiple, albeit overlapping basic mechanisms that ultimately co-ordinate their proliferative capacity, survival and epigenetic control of lineage restriction to ultimately establish non-dividing or nonviable cell states. Within the same tissue, these may also change during development and aging as well as under different conditions of homeostasis, wounding, inflammation and infection. Even at a single moment in time, there may be multiple pathways by which a single mature cell type can be generated. Moreover, the properties of ‘self-renewal' and long-term quiescence in normal cells are now known, at least as suggested by lineage tracing studies in mice, to be more broadly distributed and less rigidly associated with the capacity to regenerate the entire tissue^100^,^101^,^102^.

These issues are important because they have necessitated a modification to the historic assumption as to the singular state of ‘stem cells' even within a given tissue. Hence a greater diversity of cell types can now be envisaged as potential initial cells in which the genetic and epigenetic changes, required to generate irreversibly expanding malignant clones, might originate. As detailed below, these considerations also undermine assumptions underpinning the historic concept of a cancer stem cell, without negating it altogether—a difficult development that the field has now had to accommodate^10^.

### *De novo* human models of tumorigenesis

**Figure 2:**
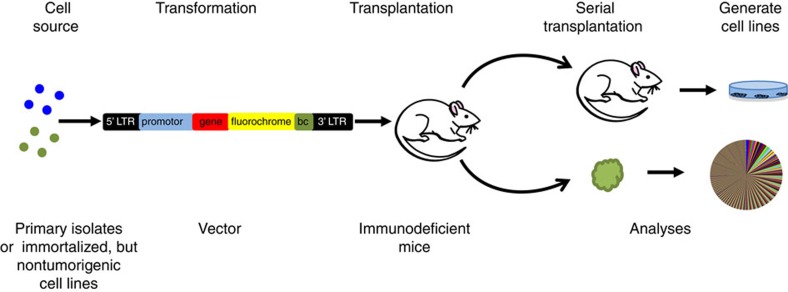
*De novo* generation of tumours from ‘normal' human cells. Most examples of successful transformation of primary sources of normal human cells (or non-tumorigenic human cell lines) have used retro- or lenti-viruses encoding one or more oncogenes and a fluorochrome (for example, GFP) to enable malignant cells to be later isolated and characterized. The transduced cells are then transplanted into a receptive site in immunodeficient mice. When a tumour forms, the cells can then be removed for morphological, immunohistochemical, flow cytomteric and/or various molecular and clonal analyses. When this method is efficient, polyclonal tumours may be generated (as illustrated by the pie chart). Retrieved viable cells can also be further transplanted or may be used to generate cell lines.

Experimental models of *de novo* tumorigenesis starting from cells isolated directly from normal human tissues are attractive because they circumvent the concerns inherent in extrapolating from immortalized cell line data or species differences (Fig. 2). However, the frequency of success has thus far been much more limited, perhaps due to a historic lag in the development of appropriate methods to isolate the relevant target cells in viable form, and/or to transduce them at an adequate efficiency. Indeed, where these issues have been carefully addressed, some models have been generated (Table 1). For example, normal human basal prostate cells transduced with a combination of vectors encoding cDNAs for *AKT*, *ERG* and the androgen receptor have been found to produce tumours in transplanted immunodeficient mice^103^. In contrast, in the same study, transplants of co-isolated luminal prostate cells transduced with the same vectors did not yield tumours. This result is interesting because the gene expression profile of prostate cancers appears closer to that of the luminal cells of the normal prostate. One explanation is that prostate cancers in which these genes are characteristically altered actually originate in basal cells that then generate progeny that have luminal features^103^. Alternatively, it may be argued that a malignant phenotype can originate *in vivo* directly in luminal cells but the conditions used to date simply fail to support this process experimentally.

*De novo* models of tumorigenesis starting from primary human cells have also now been reported for colon and mammary cells. For example, a recent study demonstrated the formation of tumours in immunodeficient mice transplanted with organoids expanded *in vitro* from colon cells in which CRISPR/Cas9 was used to generate suppressive mutations in *APC*, *SMAD4* and *TP53* and activating mutations in *KRAS* and *PIK3CA*^104^. *De novo* genesis of human breast tumours has also now been achieved in immunodeficient mice transplanted with primary isolates of normal cells transduced with *p53(R175H)*, *CCND1*, *PIK3CA* and *KRAS(G12V)*^105^, *SV40* plus *KRAS(G12V)*^106^, and more recently with just *KRAS(G12D)* alone^107^. However, in contrast to the results described for the prostate model, immunohistological analyses of these human breast tumours has indicated the presence of a mixture of phenotypes, possibly related to the polyclonal composition of the tumours generated^107^. The robustness of these models and speed of the tumorigenic process in at least some cases should make them useful for future elucidation of the minimal cellular and extrinsic factors required for their genesis.

Examples of *de novo* leukaemogenesis using primitive (CD34^+^) subsets of hematopoietic cells isolated from human cord blood is also accruing. In this case, examples of genes whose vector-mediated forced overexpression in normal cells have produced overt leukaemic populations in immunodeficient mice include cDNAs for *MLL-AF9* (refs 108, 109), *MLL-AF4* (ref. 110), *MN1* plus *NUP98HOXD13* (ref. 111), *BCR-ABL* plus a dominant-negative form of *IKAROS*^112^, and *MYC* plus *BCL*^113^. The mixed lineage leukaemia (*MLL*) gene is rearranged and fuses with multiple partner genes in both spontaneously arising human AML and acute lymphoid leukemia (ALL), but the *MLL-AF9* fusion oncogene is associated almost exclusively with AML in humans^108^. Interestingly, overexpression of *MLL-AF9* in normal cord blood cells produced ALL in transplanted Non-obese diabetic-Scid (NOD-SCID) hosts, but AML in NOD-SCID mice that had been engineered to express three human growth factors. This result illustrates the ability of external factors to dictate the phenotype of the malignant cells produced and further underscores the fallacy in assuming the predominant cell type necessarily reflects the cell of origin or the specific oncogene driving the tumorigenic state. The *MN1-NUP98HOXD13* model also serves to illustrate the difference between effects obtained in analogous mouse and human target cells. In mouse cells, *MN1* alone was sufficient to induce a myeloid leukaemia, whereas in human cord blood cells it induced only a transient myeloproliferation. Only when an activated *HOX* gene (that is, *NUP98HOXD13*), was also introduced was a serially transplantable AML obtained in the human cells.

These studies demonstrate some of the unique aspects of tumorigenesis in primary human cells that are not well predicted by mouse models. They also provide examples of experimentally generated *de novo* human tumours whose characteristics are heavily influenced by factors beyond the cell of origin, or the genetic alterations used.

### Functional diversity of human tumour subclones

Most studies attempting to analyse the subclonal diversity of malignant human populations that arise in patients have relied on retrospective inferences derived from analyses of the genomic or phenotypic characterization of primary samples and/or changes incurred in serial transplants of these cells in immunodeficient mouse hosts^23^,^114^,^115^,^116^,^117^,^118^. However, these approaches generally do not allow the frequency of clonogenic cells to be quantified, nor the definition of their genomic or phenotypic properties. These are important parameters because the genomic instability of many established neoplastic human populations can produce genetic alterations that are irrelevant to the continued growth of the tumour. Examples of such mutations have been well documented in genetic analyses of the leukemic cells from patients with chronic phase CML^119^.

Limiting dilution analysis (LDA) and vector-based tracking of clonogenic cells offer powerful approaches to quantify malignant cells with proliferative potential *in vivo*. The LDA approach has been extensively applied to a number of primary human tumour types that can engraft immunodeficient mice. These include malignant populations that arise in the human brain^47^,^120^, colon^48^,^121^, prostate^122^, breast^46^, ovary^123^, skin/melanoma^54^,^124^ and the hematopoietic system^45^,^125^,^126^,^127^,^128^. Coupling these approaches to prospectively isolated phenotypes of cells within the transplanted populations has been the basis of identifying the subset(s) of cells actually possessing the tumorigenic activity detected in the recipients. However, to exploit this approach to characterize the clonal growth properties of *single* tumorigenic cells, the transplants must be initiated with cell numbers that produce tumours at a frequency of less than one in three mice. The large numbers of mice required to obtain clonal data can thus become rapidly prohibitively expensive, if not impractical, particularly when clonal outputs may vary quantitatively as well as phenotypically and dynamically within and between different primary tumours being assessed.

The use of massively parallel DNA sequencing has improved the sensitivity and efficiency of vector insert analysis to identify clones based on their semi-random integration sites^129^, thus circumventing many of the limitations of LDA approaches. Nevertheless, ∼50% of insertion sites are still elusive to detection using the latest versions of this methodology^129^. A more recent alternative has been the creation and use of libraries of DNA barcoded lentiviral vectors^130^. When such libraries are used at a concentration that results in the transduction of less than one in three cells in the target population being interrogated, and the diversity of the library is greater than the number of cells being investigated, the likelihood that each of the initially marked cells contains a unique barcode is high. The frequency of each barcode in the DNA extracted from populations derived from barcoded cells can then be used to infer the number of cells present in different clones. For both vector strategies, phenotypic purification of the cells to be analysed before extracting the DNA for insert site or barcode analysis allows the phenotypic composition of each clone to also be characterized^109^,^131^,^132^,^133^,^134^,^135^,^136^,^137^.

Application of these protocols has revealed complex clonal dynamics in human tumours derived from serially passaged cells^131^. Interestingly, this exercise has documented highly diverse growth behaviours of single cells from cell lines when large numbers of these are transplanted, even though single-cell transplant experiments indicate more than one third can generate a tumour. Thus, to relate the growth activity of the clones with their genotype, it is necessary to isolate and sequence the individual clones generated, as has been done in human glioblastoma^138^.

## The future—new opportunities

Technological advances are well known to be the drivers of new information. Nowhere is this more relevant than to current issues of interest in the general arena of biomedicine. In the field of human cell oncogenesis, three technologies are of particular relevance: organoid cultures, reprogramming and CRISPR/Cas9. Organoid culture systems are allowing more faithful tissue development to be generated from primitive normal cells and are showing promise for supporting the expansion *in vitro* of cells derived directly from human tumours^139^,^140^,^141^. Reprogramming allows permanent clonal lines of induced pluripotent stem cells (iPSCs) to be generated from individual malignant cells. CRISPR/Cas9 enables the use of precise gene editing to create new models of human tumours and to test the role of specific genetic alterations in establishing or maintaining the transformed status of patient-derived malignant cells.

### Organoids

This term refers to 3D structures generated in suspension cultures under conditions where 2D growth of attached cells is blocked or prevented (either by suspending the cells in a semi-solid medium-like matrigel or the use of a culture container that prevents cell attachment). The generation of 3D organoids to engineer tissues that are histologically and functionally more similar to their *in vivo* counterparts will facilitate the modelling of human disorders, performing drug screens, and the creation *in vitro* of replacement tissues and/or organs. Methods combining directed differentiation of cells in culture systems that promote organoid formation are being developed for many complex tissues such as the liver, kidney, intestine, eye, and brain, to name a few^142^. The advantages of human organoid cultures include better control of the cellular milieu than is associated with tumorigenesis *in vivo*, better spatial organization of cell types than is achieved in 2D *in vitro* systems and improved mimicry of *in vivo* cell behaviour, potential for larger scale testing of variables and drug effects, and reduced ethical concerns and costs associated with animal xenograft experiments.

### Induced pluripotent stem cells

iPSC technology offers a novel method of capturing the genomic state of single cells from almost any source, including transformed cells. However, this comes at the cost of losing the malignant phenotype of the original cells from which each iPSC clone is derived. Nevertheless, from the limited experience where it has been applied to the study of cancer to date, it appears that at least some properties of the malignant cells of origin may be reactivated by stimulating the iPSCs to differentiate back into the lineage or tissue from which the malignant cells arose and may then also be used for drug screening^143^,^144^.

iPSCs have been derived from human chronic phase CML cells with documented retention and expression of the signature *BCR-ABL1* fusion gene^145^,^146^,^147^. Additional examples of human hematopoietic cells from which iPSCs (or iPSC-like cells) have been obtained include cells from patients with MDS^148^, polycythemia vera^149^ and juvenile myelomonocytic leukemia^150^, as well as originally normal human hematopoietic cells forced to overexpress *MLL*^151^. iPSCs have also been generated from human pancreatic ductal adenocarcinomas^152^, Multiple endocrine neoplasia type 2A (MEN2A)^153^, bladder cancer cell lines^154^ and cells from an individual with Li Fraumeni syndrome, a congenital cancer predisposition genotype in which one allele of the *p53* gene is mutated^155^. In this case, the iPSCs were used to study the epigenetic states of tumour formation^156^ and the bidirectional reversibility of epigenetic processes associated with iPSC reprogramming^157^.

### CRISPR/Cas9

**Figure 3:**
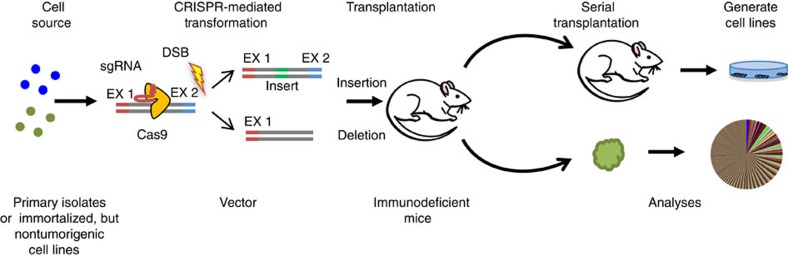
Use of> *CRISPR/Cas9* gene editing to examine the tumorigenic consequences of modifying specific genes in human cells. In this methodology, the test cells are exposed to CRISPR/Cas9 reagents and then transplanted into immunodeficient mice as in Fig. 2.

The CRISPR/Cas9 technology originates from type II CRISPR-Cas systems that evolved to provide bacteria with a form of adaptive immunity to viruses and plasmids. The CRISPR-associated Cas9 protein is an endonuclease that introduces a site-specific double-strand break (DSB) into DNA. The ability of CRISPR/Cas9 to introduce DSBs at defined positions using a guide RNA can also be used to generate human cell lines and primary cells bearing chromosomal translocations resembling those described in human cancers (Fig. 3). Examples to date include lung cancer^158^, AML^159^ and Ewing's sarcoma^160^. An improved method to generate models of liver cancer or myeloid malignancy in mice using CRISPR/Cas9 was also recently reported^161^,^162^. Combining CRISPR/Cas9 with barcoding has been used to track the clonal dynamics of lung cancer cells during their acquired resistance to EGFR inhibitors and subsequent response to combined drug therapies^163^,^164^. Combining the generation of iPSCs with CRISPR/Cas9 holds the future possibility of both creating new models of human cancer and of testing the ultimate therapeutic potential of gene-targeting approaches.

## Summary

Recreating the process of human tumorigenesis in naive human cells has been a challenging frontier not anticipated by the historically contrasting ease of achieving this outcome in mouse cells. However, several major developments are now changing this picture. One has been the generation of long-lived mice in which multiple components of the innate and acquired immune system have been suppressed. This advance now allows transformed human cells to grow and evolve into fully malignant populations in an *in vivo* setting. Together with improvements in lentiviral design and production, and methods to isolate and purify normal viable human cells, these advances are now enabling a growing number of *de novo* models of human tumorigenesis to be developed. The use of DNA barcoding of the initial cells is now also providing a tractable method to analyse the clonal composition and dynamics of the malignant cells generated and should provide a powerful approach to examine therapeutic effects in the future. These approaches coupled with emerging methods to modify and regulate cell behaviour even more precisely herald a new era of improved understanding of the complex processes of human tumorigenesis.

## Additional information

**How to cite this article:** Balani, S. *et al*. Modeling the process of human tumorigenesis. *Nat. Commun.* **8**, 15422 doi: 10.1038/ncomms15422 (2017).

**Publisher's note:** Springer Nature remains neutral with regard to jurisdictional claims in published maps and institutional affiliations.

## Figures and Tables

**Table 1:** Examples of *de novo* tumour models from primary human cell sources.

**Human Tissue**	**Gene**	**Finding**	**Reference**
Prostate	*AKT, ERG, AR*	Tumour formation in basal cells but not in luminal cells.	103
Colon	*APC, SMAD4, TP53, KRAS, PIK3CA*	Organoids engineered to express all five mutations grew independently of niche factors *in vitro*, and could be transplanted to form tumours in mice.	104
Mammary	*p53(R175H), CCND1, PIK3CA,* and *KRAS(G12V)*	Cells with BRCA1-mutation form tumours and showed increased basal differentiation compared to cells from non-carrier tissues. EpCAM+CD10− luminal cells from both BRCA1+/+ and BRCA1mut/+ tissues were enriched for tumour-forming ability.	105
	*SV40* and *KRAS(G12V)*	Transformation of EpCAM+ cells resulted in the development of common forms of breast cancer, including ER+ and ER− tumours with luminal and basal-like characteristics, respectively. Transformation of CD10+ (basal) cells resulted in the development of rare metaplastic tumours similar to the claudin-low subtype.	106
	*KRAS(G12D)*	Both basal and luminal cells generated polyclonal serial transplantable tumours containing a mixture of phenotypes and clones with variable growth dynamics revealed in serial transplants.	107
Blood	*MLL-AF9*	Some leukemia stem cells (LSCs) were multi-potent and could be lineage directed by altering either the growth factors or the recipient strain of mouse, highlighting the importance of microenvironment. Other LSCs were strictly lineage committed, demonstrating the heterogeneity of the stem cell compartment in the MLL diseases produced.	108,109
	*MLL-AF4*	Generation of a model of t(4;11) pro-B ALL that fully recapitulated the immunophenotypic and molecular features of the disease that appears in patients.	110
	*MN1* and *NUP98HOXD13*	Co-transduction of an activated HOX gene (NUP98HOXD13) with MN1 induced a serially transplantable AML.	111
	*BCR-ABL1* and *dnIKAROS (IK6)*	An aggressive AML with disseminated myeloid sarcomas developed within 4 weeks following transplantation of cord blood cells transduced with both genes.	112
	*MYC and BCL2*	Production of a model of lymphoma that recapitulates the histopathological and clinical aspects of steroid-, chemotherapy- and rituximab-resistant human ‘double-hit' MYC-BCL2 lymphoma.	113
	*DEK-NUP214*	Development of a human cell AML with phenotypic characteristics of a t(6;9) disease and CD45+CD13+CD34+CD38+ immunophenotype.	165
	*ZMYM2-FGFR1*	Development of myeloproliferative disease that progresses to AML. Mice show hepatospenomegaly, hypercellular bone marrow and a CD45+CD34+CD13+ immunophenotype.	166

## References

[b1] Hanahan D. & Weinberg R. A. Hallmarks of cancer: the next generation. Cell 144, 646–674 (2011). 21376230 10.1016/j.cell.2011.02.013

[b2] Atlas T. C. G. *TCGA Research Network Publications.* Available at: https://cancergenome.nih.gov/publications.

[b3] Friedman J. M. & Fialkow P. J. Cell marker studies of human tumorigenesis. Transplant. Rev. 28, 17–33 (1976). 766326 10.1111/j.1600-065x.1976.tb00190.x

[b4] Nachman M. W. & Crowell S. L. Estimate of the mutation rate per nucleotide in humans. Genetics 156, 297–304 (2000). 10978293 10.1093/genetics/156.1.297 PMC1461236

[b5] Whiteman D. C. & Wilson L. F. The fractions of cancer attributable to modifiable factors: A global review. Cancer Epidemiol. 44, 203–221 (2016). 27460784 10.1016/j.canep.2016.06.013

[b6] Sitas F. Twenty five years since the first prospective study by Forman *et al*. (1991) on Helicobacter pylori and stomach cancer risk. Cancer Epidemiol. 41, 159–164 (2016). 26922171 10.1016/j.canep.2016.02.002

[b7] Grulich A. E., van Leeuwen M. T., Falster M. O. & Vajdic C. M. Incidence of cancers in people with HIV/AIDS compared with immunosuppressed transplant recipients: a meta-analysis. Lancet (London) 370, 59–67 (2007). 10.1016/S0140-6736(07)61050-2 17617273

[b8] McMahon B. . Hepatitis-associated liver cancer: gaps and opportunities to improve care. J. Natl Cancer. Inst. 108, 4 doi: 10.1093/jnci/djv359 (2016). 10.1093/jnci/djv359 PMC4857154 26626106

[b9] Brock A., Krause S. & Ingber D. E. Control of cancer formation by intrinsic genetic noise and microenvironmental cues. Nat. Rev. Cancer 15, 499–509 (2015). 26156637 10.1038/nrc3959

[b10] Rosenbloom D. I., Camara P. G., Chu T. & Rabadan R. Evolutionary scalpels for dissecting tumor ecosystems. Biochim. Biophys. Acta doi: 10.1016/j.bbcan.2016.11.005 (2016). 10.1016/j.bbcan.2016.11.005 PMC5704952 27923679

[b11] Nowell P. C. The clonal evolution of tumor cell populations. Science 194, 23–28 (1976). 959840 10.1126/science.959840

[b12] Fearon E. R. & Vogelstein B. A genetic model for colorectal tumorigenesis. Cell 61, 759–767 (1990). 2188735 10.1016/0092-8674(90)90186-i

[b13] Steensma D. P. . Clonal hematopoiesis of indeterminate potential and its distinction from myelodysplastic syndromes. Blood 126, 9–16 (2015). ***This report summarizes data from very recent studies documenting an increasing age-associated appearance of oncogenic mutations in amplified clones of normally differentiating cells without any evidence of immediate or necessary progression to malignancy. These findings established the concept of clonal evolution preceding acquisition of a fully malignant state in the blood-forming system where abnormal cell distributions and arrangements in situ are difficult to discern***. 25931582 10.1182/blood-2015-03-631747 PMC4624443

[b14] Martincorena I. . Tumor evolution. High burden and pervasive positive selection of somatic mutations in normal human skin. Science 348, 880–886 (2015). 25999502 10.1126/science.aaa6806 PMC4471149

[b15] Blokzijl F. . Tissue-specific mutation accumulation in human adult stem cells during life. Nature 538, 260–264 (2016). 27698416 10.1038/nature19768 PMC5536223

[b16] Negrini S., Gorgoulis V. G. & Halazonetis T. D. Genomic instability [mdash] an evolving hallmark of cancer. Nat. Rev. Mol. Cell. Biol. 11, 220–228 (2010). 20177397 10.1038/nrm2858

[b17] Choi J. D. & Lee J. S. Interplay between Epigenetics and Genetics in cancer. Genomics Inform. 11, 164–173 (2013). 24465226 10.5808/GI.2013.11.4.164 PMC3897842

[b18] Avgustinova A. & Benitah S. A. The epigenetics of tumour initiation: cancer stem cells and their chromatin. Curr. Opin. Genet. Dev. 36, 8–15 (2016). 26874045 10.1016/j.gde.2016.01.003

[b19] Lee J. K., Choi Y. L., Kwon M. & Park P. J. Mechanisms and consequences of cancer genome instability: lessons from genome sequencing studies. Annu. Rev. Pathol. 11, 283–312 (2016). 26907526 10.1146/annurev-pathol-012615-044446

[b20] Notta F. . A renewed model of pancreatic cancer evolution based on genomic rearrangement patterns. Nature 538, 378–382 (2016). ***This study was the first to challenge the generally accepted model of how a solid tumour develops. Through a detailed genomic analysis of patients' samples the authors obtained evidence of a sudden burst of important mutational events rather than a slow stepwise accumulation of driving mutations***. 27732578 10.1038/nature19823 PMC5446075

[b21] Aparicio S. & Caldas C. The implications of clonal genome evolution for cancer medicine. N. Engl. J. Med. 368, 842–851 (2013). 23445095 10.1056/NEJMra1204892

[b22] Nik-Zainal S. . Landscape of somatic mutations in 560 breast cancer whole-genome sequences. Nature 534, 47–54 (2016). 27135926 10.1038/nature17676 PMC4910866

[b23] Shlush L. I. . Identification of pre-leukaemic haematopoietic stem cells in acute leukaemia. Nature 506, 328–333 (2014). 24522528 10.1038/nature13038 PMC4991939

[b24] Qian M., Wang D. C., Chen H. & Cheng Y. Detection of single cell heterogeneity in cancer. Semin. Cell Dev. Biol. 64, 143–149 (2016). 27619166 10.1016/j.semcdb.2016.09.003

[b25] Sandberg R. Entering the era of single-cell transcriptomics in biology and medicine. Nat. Methods 11, 22–24 (2014). 24524133 10.1038/nmeth.2764

[b26] Curtis C. . The genomic and transcriptomic architecture of 2,000 breast tumours reveals novel subgroups. Nature 486, 346–352 (2012). 22522925 10.1038/nature10983 PMC3440846

[b27] Blanpain C. Tracing the cellular origin of cancer. Nat. Cell Biol. 15, 126–134 (2013). 23334500 10.1038/ncb2657

[b28] Kucherlapati R. Genetically modified mouse models for biomarker discovery and preclinical drug testing. Clin. Cancer Res. 18, 625–630 (2012). 22298895 10.1158/1078-0432.CCR-11-2021

[b29] Becher O. J. & Holland E. C. Genetically engineered models have advantages over xenografts for preclinical studies. Cancer Res. 66, 3355–3358, discussion 3358–3359 (2006). 16585152 10.1158/0008-5472.CAN-05-3827

[b30] Ashworth A., Lord C. J. & Reis-Filho J. S. Genetic interactions in cancer progression and treatment. Cell 145, 30–38 (2011). 21458666 10.1016/j.cell.2011.03.020

[b31] Shi Y. . Chromatin accessibility contributes to simultaneous mutations of cancer genes. Sci. Rep. 6, 35270 (2016). 27762310 10.1038/srep35270 PMC5071887

[b32] Narayan S., Bader G. D. & Reimand J. Frequent mutations in acetylation and ubiquitination sites suggest novel driver mechanisms of cancer. Genome Med. 8, 55 (2016). 27175787 10.1186/s13073-016-0311-2 PMC4864925

[b33] Alexandrov L. B. . Signatures of mutational processes in human cancer. Nature 500, 415–421 (2013). 23945592 10.1038/nature12477 PMC3776390

[b34] Petzer A. . Characterization of primitive subpopulations of normal and leukemic cells present in the blood of patients with newly diagnosed as well as established chronic myeloid leukemia. Blood 88, 2162–2171 (1996). 8822936

[b35] Eisterer W. . Different subsets of primary chronic myeloid leukemia stem cells engraft immunodeficient mice and produce a model of the human disease. Leukemia 19, 435–441 (2005). 15674418 10.1038/sj.leu.2403649

[b36] Chu S. . Persistence of leukemia stem cells in chronic myelogenous leukemia patients in prolonged remission with imatinib treatment. Blood 118, 5565–5572 (2011). 21931114 10.1182/blood-2010-12-327437 PMC3217358

[b37] Pellicano F., Mukherjee L. & Holyoake T. L. Concise review: cancer cells escape from oncogene addiction: understanding the mechanisms behind treatment failure for more effective targeting. Stem Cells 32, 1373–1379 (2014). 24520002 10.1002/stem.1678

[b38] Fearon E. R., Burke P. J., Schiffer C. A., Zehnbauer B. A. & Vogelstein B. Differentiation of leukemia cells to polymorphonuclear leukocytes in patients with acute nonlymphocytic leukemia. N. Engl. J. Med. 315, 15–24 (1986). 3086723 10.1056/NEJM198607033150103

[b39] Eppert K. . Stem cell gene expression programs influence clinical outcome in human leukemia. Nat. Med. 17, 1086–1093 (2011). 21873988 10.1038/nm.2415

[b40] Jan M. . Clonal evolution of preleukemic hematopoietic stem cells precedes human acute myeloid leukemia. Sci. Transl. Med. 4, 149ra118 (2012). 10.1126/scitranslmed.3004315 PMC4045621 22932223

[b41] Corces-Zimmerman M. R., Hong W. J., Weissman I. L., Medeiros B. C. & Majeti R. Preleukemic mutations in human acute myeloid leukemia affect epigenetic regulators and persist in remission. Proc. Natl Acad. Sci. USA 111, 2548–2553 (2014). 24550281 10.1073/pnas.1324297111 PMC3932921

[b42] Smith F. O., Raskind W. H., Fialkow P. J. & Bernstein I. D. Cellular biology of acute myelogenous leukemia. J. Pediatr. Hematol. Oncol. 17, 113–122 (1995). 7749760 10.1097/00043426-199505000-00004

[b43] Prchal J. T. . A common progenitor for human myeloid and lymphoid cells. Nature 274, 590–591 (1978). 672990 10.1038/274590a0

[b44] Raskind W. H., Tirumali N., Jacobson R., Singer J. & Fialkow P. J. Evidence for a multistep pathogenesis of a myelodysplastic syndrome. Blood 63, 1318–1323 (1984). 6326894

[b45] Bonnet D. & Dick J. E. Human acute myeloid leukemia is organized as a hierarchy that originates from a primitive hematopoietic cell. Nat. Med. 3, 730–737 (1997). ***This study was the first to demonstrate that human cells able to regenerate leukemic populations in vivo(in a transplanted, sublethally irradiated immunodeficient mouse) are restricted to the CD34+ subset and that includes cells with serially transplantable leukemia propagating activity indicative of leukemic stem cell activity***. 9212098 10.1038/nm0797-730

[b46] Al-Hajj M., Wicha M. S., Benito-Hernandez A., Morrison S. J. & Clarke M. F. Prospective identification of tumorigenic breast cancer cells. Proc. Natl Acad. Sci. USA 100, 3983–3988 (2003). 12629218 10.1073/pnas.0530291100 PMC153034

[b47] Singh S. K. . Identification of human brain tumour initiating cells. Nature 432, 396–401 (2004). 15549107 10.1038/nature03128

[b48] O'Brien C. A., Pollett A., Gallinger S. & Dick J. E. A human colon cancer cell capable of initiating tumour growth in immunodeficient mice. Nature 445, 106–110 (2007). 17122772 10.1038/nature05372

[b49] Holyoake T., Jiang X., Eaves C. & Eaves A. Isolation of a highly quiescent subpopulation of primitive leukemic cells in chronic myeloid leukemia. Blood 94, 2056–2064 (1999). ***This study was the first to document the presence of a quiescent population of malignant cells setting the stage for understanding why anti-proliferative agents are not effective longterm in eradicating many human cancers***. 10477735

[b50] Gao M. Q., Choi Y. P., Kang S., Youn J. H. & Cho N. H. CD24+ cells from hierarchically organized ovarian cancer are enriched in cancer stem cells. Oncogene 29, 2672–2680 (2010). 20190812 10.1038/onc.2010.35

[b51] Pece S. . Biological and molecular heterogeneity of breast cancers correlates with their cancer stem cell content. Cell 140, 62–73 (2010). 20074520 10.1016/j.cell.2009.12.007

[b52] Roesch A. . A temporarily distinct subpopulation of slow-cycling melanoma cells is required for continuous tumor growth. Cell 141, 583–594 (2010). 20478252 10.1016/j.cell.2010.04.020 PMC2882693

[b53] Dembinski J. L. & Krauss S. Characterization and functional analysis of a slow cycling stem cell-like subpopulation in pancreas adenocarcinoma. Clin. Exp. Metastasis 26, 611 (2009). 19421880 10.1007/s10585-009-9260-0 PMC2776152

[b54] Quintana E. . Efficient tumour formation by single human melanoma cells. Nature 456, 593–598 (2008). ***This study was the first to show that in many human melanomas almost every cell has the innate ability to regenerate a tumour, thus providing an important example of a human tumour that does not fit a stem cell model***. 19052619 10.1038/nature07567 PMC2597380

[b55] Liu S. . Breast cancer stem cells transition between epithelial and mesenchymal states reflective of their normal counterparts. Stem Cell Rep. 2, 78–91 (2014). 10.1016/j.stemcr.2013.11.009 PMC3916760 24511467

[b56] Friedl P. & Alexander S. Cancer invasion and the microenvironment: plasticity and reciprocity. Cell 147, 992–1009 (2011). 22118458 10.1016/j.cell.2011.11.016

[b57] Chaffer C. L., San Juan B. P., Lim E. & Weinberg R. A. EMT, cell plasticity and metastasis. Cancer Metastasis Rev. 35, 645–654 (2016). 27878502 10.1007/s10555-016-9648-7

[b58] Lehuede C., Dupuy F., Rabinovitch R., Jones R. G. & Siegel P. M. Metabolic plasticity as a determinant of tumor growth and metastasis. Cancer Res. 76, 5201–5208 (2016). 27587539 10.1158/0008-5472.CAN-16-0266

[b59] Beer P. A. & Eaves C. J. Modeling normal and disordered human hematopoiesis. Trends in Cancer 1, 199–210 (2016). 10.1016/j.trecan.2015.09.002 28741474

[b60] Liu X. . Low CD38 identifies progenitor-like inflammation-associated luminal cells that can initiate human prostate cancer and predict poor outcome. Cell Rep. 17, 2596–2606 (2016). 27926864 10.1016/j.celrep.2016.11.010 PMC5367888

[b61] Yoo Y. A. . Bmi1 marks distinct castration-resistant luminal progenitor cells competent for prostate regeneration and tumour initiation. Nat. Commun. 7, 12943 (2016). 27703144 10.1038/ncomms12943 PMC5059479

[b62] Shin S. . Genetic lineage tracing analysis of the cell of origin of hepatotoxin-induced liver tumors in mice. Hepatology (Baltimore) 64, 1163–1177 (2016). 27099001 10.1002/hep.28602 PMC5033674

[b63] Leeman K. T., Fillmore C. M. & Kim C. F. Lung stem and progenitor cells in tissue homeostasis and disease. Curr. Top. Dev. Biol. 107, 207–233 (2014). 24439808 10.1016/B978-0-12-416022-4.00008-1 PMC4038302

[b64] Van Keymeulen A. . Reactivation of multipotency by oncogenic PIK3CA induces breast tumour heterogeneity. Nature 525, 119–123 (2015). 26266985 10.1038/nature14665

[b65] Koren S. . PIK3CA(H1047R) induces multipotency and multi-lineage mammary tumours. Nature 525, 114–118 (2015). 26266975 10.1038/nature14669

[b66] Makarem M. . Developmental changes in the in vitro activated regenerative activity of primitive mammary epithelial cells. PLoS Biol. 11, e1001630 (2013). 23966837 10.1371/journal.pbio.1001630 PMC3742452

[b67] Stingl J. . Purification and unique properties of mammary epithelial stem cells. Nature 439, 993–997 (2006). 16395311 10.1038/nature04496

[b68] Shackleton M. . Generation of a functional mammary gland from a single stem cell. Nature 439, 84–88 (2006). 16397499 10.1038/nature04372

[b69] Meyer D. S. . Luminal expression of PIK3CA mutant H1047R in the mammary gland induces heterogeneous tumors. Cancer Res. 71, 4344–4351 (2011). 21482677 10.1158/0008-5472.CAN-10-3827

[b70] Melchor L. . Identification of cellular and genetic drivers of breast cancer heterogeneity in genetically engineered mouse tumour models. J. Pathol. 233, 124–137 (2014). 24615332 10.1002/path.4345

[b71] Powell A. E. . Inducible loss of one Apc allele in Lrig1-expressing progenitor cells results in multiple distal colonic tumors with features of familial adenomatous polyposis. Am. J. Physiol. Gastrointest. Liver Physiol. 307, G16–G23 (2014). 24833705 10.1152/ajpgi.00358.2013 PMC4080164

[b72] Boumahdi S. . SOX2 controls tumour initiation and cancer stem-cell functions in squamous-cell carcinoma. Nature 511, 246–250 (2014). 24909994 10.1038/nature13305

[b73] Vanner R. J. . Quiescent sox2(+) cells drive hierarchical growth and relapse in sonic hedgehog subgroup medulloblastoma. Cancer Cell 26, 33–47 (2014). 24954133 10.1016/j.ccr.2014.05.005 PMC4441014

[b74] Larsimont J. C. . Sox9 Controls Self-Renewal of Oncogene Targeted Cells and Links Tumor Initiation and Invasion. Cell Stem Cell 17, 60–73 (2015). 26095047 10.1016/j.stem.2015.05.008

[b75] Schwitalla S. . Intestinal tumorigenesis initiated by dedifferentiation and acquisition of stem-cell-like properties. Cell 152, 25–38 (2013). 23273993 10.1016/j.cell.2012.12.012

[b76] Moller Y. . Oncogenic Ras triggers hyperproliferation and impairs polarized colonic morphogenesis by autocrine ErbB3 signaling. Oncotarget 7, 53526–53539 (2016). 27447549 10.18632/oncotarget.10658 PMC5288203

[b77] Malaterre J. . Intestinal-specific activatable Myb initiates colon tumorigenesis in mice. Oncogene 35, 2475–2484 (2016). 26300002 10.1038/onc.2015.305 PMC4867492

[b78] Bellafante E. . PGC-1beta promotes enterocyte lifespan and tumorigenesis in the intestine. Proc. Natl Acad. Sci. USA 111, E4523–E4531 (2014). 25288742 10.1073/pnas.1415279111 PMC4210309

[b79] Pineault N. . Induction of acute myeloid leukemia in mice by the human leukemia-specific fusion gene NUP98-HOXD13 in concert with Meis1. Blood 101, 4529–4538 (2003). 12543865 10.1182/blood-2002-08-2484

[b80] Asai T. . Generation of a novel, multi-stage, progressive, and transplantable model of plasma cell neoplasms. Sci. Rep. 6, 22760 (2016). 26961797 10.1038/srep22760 PMC4785351

[b81] Scourzic L. . DNMT3A(R882H) mutant and Tet2 inactivation cooperate in the deregulation of DNA methylation control to induce lymphoid malignancies in mice. Leukemia 30, 1388–1398 (2016). 26876596 10.1038/leu.2016.29 PMC4869893

[b82] Rappaport A. & Johnson L. Genetically engineered knock-in and conditional knock-in mouse models of cancer. Cold Spring Harb. Protoc. 2014, 897–911 (2014). 25183823 10.1101/pdb.top069799

[b83] Yi L. & Li J. CRISPR-Cas9 therapeutics in cancer: promising strategies and present challenges. Biochim. Biophys. Acta 1866, 197–207 (2016). 27641687 10.1016/j.bbcan.2016.09.002

[b84] Kannan R. & Ventura A. The CRISPR revolution and its impact on cancer research. Swiss Med. Wkly 145, w14230 (2015). 26661454 10.4414/smw.2015.14230 PMC5512432

[b85] Annunziato S. . Modeling invasive lobular breast carcinoma by CRISPR/Cas9-mediated somatic genome editing of the mammary gland. Genes Dev. 30, 1470–1480 (2016). 27340177 10.1101/gad.279190.116 PMC4926868

[b86] Wasylishen A. R. . New model systems provide insights into Myc-induced transformation. Oncogene 30, 3727–3734 (2011). 21441954 10.1038/onc.2011.88 PMC3765156

[b87] Bessette D. C. . Using the MCF10A/MCF10CA1a breast cancer progression cell line model to investigate the effect of active, mutant forms of EGFR in breast cancer development and treatment using gefitinib. PLoS ONE 10, e0125232 (2015). 25969993 10.1371/journal.pone.0125232 PMC4430383

[b88] Lombardo Y. . Nicastrin regulates breast cancer stem cell properties and tumor growth in vitro and in vivo. Proc. Natl Acad. Sci. USA 109, 16558–16563 (2012). 23012411 10.1073/pnas.1206268109 PMC3478621

[b89] Ward T. M. . Truncated p110 ERBB2 induces mammary epithelial cell migration, invasion and orthotopic xenograft formation, and is associated with loss of phosphorylated STAT5. Oncogene 32, 2463–2474 (2013). 22751112 10.1038/onc.2012.256 PMC3655379

[b90] Palafox M. . RANK induces epithelial-mesenchymal transition and stemness in human mammary epithelial cells and promotes tumorigenesis and metastasis. Cancer Res. 72, 2879–2888 (2012). 22496457 10.1158/0008-5472.CAN-12-0044

[b91] Mullins S. R. . Three-dimensional cultures modeling premalignant progression of human breast epithelial cells: role of cysteine cathepsins. Biol. Chem. 393, 1405–1416 (2012). 23667900 10.1515/hsz-2012-0252 PMC3789365

[b92] Qu Y. . Generation of prostate tumor-initiating cells is associated with elevation of reactive oxygen species and IL-6/STAT3 signaling. Cancer Res. 73, 7090–7100 (2013). 24101153 10.1158/0008-5472.CAN-13-1560

[b93] Pellacani D. . Analysis of normal human mammary epigenomes reveals cell-specific active enhancer states and associated transcription factor networks. Cell Rep. 17, 2060–2074 (2016). 27851968 10.1016/j.celrep.2016.10.058

[b94] Finak G. . Stromal gene expression predicts clinical outcome in breast cancer. Nat. Med. 14, 518–527 (2008). 18438415 10.1038/nm1764

[b95] Wu J., Liang C., Chen M. & Su W. Association between tumor-stroma ratio and prognosis in solid tumor patients: a systematic review and meta-analysis. Oncotarget 7, 68954–68965 (2016). 27661111 10.18632/oncotarget.12135 PMC5356603

[b96] Lee D. . Intratumor stromal proportion predicts aggressive phenotype of gastric signet ring cell carcinomas. Gastric Cancer 1–11 doi:; DOI: 10.1007/s10120-016-0669-2 (2016). 27858181

[b97] Quail D. F. & Joyce J. A. Microenvironmental regulation of tumor progression and metastasis. Nat. Med. 19, 1423–1437 (2013). 24202395 10.1038/nm.3394 PMC3954707

[b98] McAllister S. S. & Weinberg R. A. The tumour-induced systemic environment as a critical regulator of cancer progression and metastasis. Nat. Cell Biol. 16, 717–727 (2014). 25082194 10.1038/ncb3015 PMC6220424

[b99] Valent P. . Cancer stem cell definitions and terminology: the devil is in the details. Nat. Rev. Cancer 12, 767–775 (2012). 23051844 10.1038/nrc3368

[b100] Wuidart A. . Quantitative lineage tracing strategies to resolve multipotency in tissue-specific stem cells. Genes Dev. 30, 1261–1277 (2016). 27284162 10.1101/gad.280057.116 PMC4911926

[b101] Beumer J. & Clevers H. Regulation and plasticity of intestinal stem cells during homeostasis and regeneration. Development (Cambridge) 143, 3639–3649 (2016). 10.1242/dev.133132 27802133

[b102] Chen F. & Fine A. Stem Cells in Lung Injury and Repair. Am. J. Pathol. 186, 2544–2550 (2016). 27524796 10.1016/j.ajpath.2016.05.023 PMC5222968

[b103] Goldstein A. S., Huang J., Guo C., Garraway I. P. & Witte O. N. Identification of a cell of origin for human prostate cancer. Science 329, 568–571 (2010). ***This study was the first toproduce human prostate cancers de novo and show that the histological characterization of cancers does not necessarily correlate with the cellular origin of the malignant clone***. 20671189 10.1126/science.1189992 PMC2917982

[b104] Matano M. . Modeling colorectal cancer using CRISPR-Cas9-mediated engineering of human intestinal organoids. Nat. Med. 21, 256–262 (2015). ***This study was the first study to combine the use of CRISPR-Cas9 and organoid technologies s to engineer the generation of colorectal cancer from normal human intenstinal epithelium***. 25706875 10.1038/nm.3802

[b105] Proia T. A. . Genetic predisposition directs breast cancer phenotype by dictating progenitor cell fate. Cell Stem Cell 8, 149–163 (2011). 21295272 10.1016/j.stem.2010.12.007 PMC3050563

[b106] Keller P. J. . Defining the cellular precursors to human breast cancer. Proc. Natl Acad. Sci. USA 109, 2772–2777 (2012). 21940501 10.1073/pnas.1017626108 PMC3286919

[b107] Nguyen L. V. . Barcoding reveals complex clonal dynamics of de novo transformed human mammary cells. Nature 528, 267–271 (2015). ***This study presented the first evidence that a single oncogene (KRAS-G12D) when introduced into either normal human mammary basal or luminal cells could force them to produce invasive tumours in transplanted immunodeficient mice. This study also revealed that serially transplantable clones were not predominant in the primary tumours revealing previously unknown cellular dynamics in the process of carcinogenesis***. 26633636 10.1038/nature15742

[b108] Wei J. . Microenvironment determines lineage fate in a human model of MLL-AF9 leukemia. Cancer Cell 13, 483–495 (2008). 18538732 10.1016/j.ccr.2008.04.020 PMC2486365

[b109] Barabe F., Kennedy J. A., Hope K. J. & Dick J. E. Modeling the initiation and progression of human acute leukemia in mice. Science 316, 600–604 (2007). 17463288 10.1126/science.1139851

[b110] Lin S. . Instructive role of MLL-fusion proteins revealed by a model of t(4;11) pro-B acute lymphoblastic leukemia. Cancer Cell 30, 737–749 (2016). 27846391 10.1016/j.ccell.2016.10.008

[b111] Imren S. . Modeling de novo leukemogenesis from human cord blood with MN1 and NUP98HOXD13. Blood 124, 3608–3612 (2014). 25339361 10.1182/blood-2014-04-564666 PMC4256911

[b112] Theocharides A. P. . Dominant-negative Ikaros cooperates with BCR-ABL1 to induce human acute myeloid leukemia in xenografts. Leukemia 29, 177–187 (2015). 24791856 10.1038/leu.2014.150

[b113] Leskov I. . Rapid generation of human B-cell lymphomas via combined expression of Myc and Bcl2 and their use as a preclinical model for biological therapies. Oncogene 32, 1066–1072 (2013). 22484426 10.1038/onc.2012.117 PMC4117216

[b114] Eirew P. . Dynamics of genomic clones in breast cancer patient xenografts at single-cell resolution. Nature 518, 422–426 (2015). 25470049 10.1038/nature13952 PMC4864027

[b115] Bruna A. . A Biobank of breast cancer explants with preserved intra-tumor heterogeneity to screen anticancer compounds. Cell 167, 260–274.e222 (2016). 27641504 10.1016/j.cell.2016.08.041 PMC5037319

[b116] Navin N. . Inferring tumor progression from genomic heterogeneity. Genome Res. 20, 68–80 (2010). 19903760 10.1101/gr.099622.109 PMC2798832

[b117] Bashashati A. . Distinct evolutionary trajectories of primary high-grade serous ovarian cancers revealed through spatial mutational profiling. J. Pathol. 231, 21–34 (2013). 23780408 10.1002/path.4230 PMC3864404

[b118] Gerlinger M. . Intratumor heterogeneity and branched evolution revealed by multiregion sequencing. N. Engl. J. Med. 366, 883–892 (2012). 22397650 10.1056/NEJMoa1113205 PMC4878653

[b119] Jiang X., Saw K. M., Eaves A. & Eaves C. Instability of BCR-ABL gene in primary and cultured chronic myeloid leukemia stem cells. J. Natl Cancer. Inst. 99, 680–693 (2007). 17470736 10.1093/jnci/djk150

[b120] Bao S. . Glioma stem cells promote radioresistance by preferential activation of the DNA damage response. Nature 444, 756–760 (2006). 17051156 10.1038/nature05236

[b121] Ricci-Vitiani L. . Identification and expansion of human colon-cancer-initiating cells. Nature 445, 111–115 (2007). 17122771 10.1038/nature05384

[b122] Collins A. T., Berry P. A., Hyde C., Stower M. J. & Maitland N. J. Prospective identification of tumorigenic prostate cancer stem cells. Cancer Res. 65, 10946–10951 (2005). 16322242 10.1158/0008-5472.CAN-05-2018

[b123] Suzuki S. . Identification and characterization of cancer stem cells in ovarian yolk sac tumors. Cancer Sci. 101, 2179–2185 (2010). 20804503 10.1111/j.1349-7006.2010.01672.x PMC11159821

[b124] Schatton T. . Identification of cells initiating human melanomas. Nature 451, 345–U311 (2008). 18202660 10.1038/nature06489 PMC3660705

[b125] Lapidot T. . A cell initiating human acute myeloid-leukemia after transplantation into Scid mice. Nature 367, 645–648 (1994). 7509044 10.1038/367645a0

[b126] Sarry J. E. . Human acute myelogenous leukemia stem cells are rare and heterogeneous when assayed in NOD/SCID/IL2R gamma c-deficient mice. J. Clin. Invest. 121, 384–395 (2011). 21157036 10.1172/JCI41495 PMC3007135

[b127] Notta F. . Evolution of human BCR-ABL1 lymphoblastic leukaemia-initiating cells. Nature 469, 362–367 (2011). 21248843 10.1038/nature09733

[b128] Ebinger S. . Characterization of rare, dormant, and therapy-resistant cells in acute lymphoblastic leukemia. Cancer Cell 30, 849–862 (2016). 27916615 10.1016/j.ccell.2016.11.002 PMC5156313

[b129] Bystrykh L. V., Verovskaya E., Zwart E., Broekhuis M. & de Haan G. Counting stem cells: methodological constraints. Nat. Methods 9, 567–574 (2012). 22669654 10.1038/nmeth.2043

[b130] Bystrykh L. V. & Belderbos M. E. Clonal analysis of cells with cellular barcoding: when numbers and sizes matter. Methods Mol. Biol. 1516, 57–89 (2016). 27044044 10.1007/7651_2016_343

[b131] Nguyen L. V. . DNA barcoding reveals diverse growth kinetics of human breast tumour subclones in serially passaged xenografts. Nat. Commun. 5, 5871 (2014). 25532760 10.1038/ncomms6871 PMC4284657

[b132] Bhang H. E. . Studying clonal dynamics in response to cancer therapy using high-complexity barcoding. Nat. Med. 21, 440–448 (2015). 25849130 10.1038/nm.3841

[b133] Klauke K. . Tracing dynamics and clonal heterogeneity of Cbx7-induced leukemic stem cells by cellular barcoding. Stem Cell Rep. 4, 74–89 (2015). 10.1016/j.stemcr.2014.10.012 PMC4297865 25434821

[b134] Wu C. . Clonal tracking of rhesus macaque hematopoiesis highlights a distinct lineage origin for natural killer cells. Cell Stem Cell 14, 486–499 (2014). 24702997 10.1016/j.stem.2014.01.020 PMC3979461

[b135] Cheung A. M. . Analysis of the clonal growth and differentiation dynamics of primitive barcoded human cord blood cells in NSG mice. Blood 122, 3129–3137 (2013). 24030380 10.1182/blood-2013-06-508432 PMC3814730

[b136] Verovskaya E. . Heterogeneity of young and aged murine hematopoietic stem cells revealed by quantitative clonal analysis using cellular barcoding. Blood 122, 523–532 (2013). 23719303 10.1182/blood-2013-01-481135

[b137] Naik S. H. . Diverse and heritable lineage imprinting of early haematopoietic progenitors. Nature 496, 229–232 (2013). 23552896 10.1038/nature12013

[b138] Meyer M. . Single cell-derived clonal analysis of human glioblastoma links functional and genomic heterogeneity. Proc. Natl Acad. Sci. USA 112, 851–856 (2015). 25561528 10.1073/pnas.1320611111 PMC4311802

[b139] Clevers H. Modeling development and disease with organoids. Cell 165, 1586–1597 (2016). 27315476 10.1016/j.cell.2016.05.082

[b140] Weeber F. . Preserved genetic diversity in organoids cultured from biopsies of human colorectal cancer metastases. Proc. Natl Acad. Sci. USA 112, 13308–13311 (2015). 26460009 10.1073/pnas.1516689112 PMC4629330

[b141] Baker L. A., Tiriac H., Clevers H. & Tuveson D. A. Modeling pancreatic cancer with organoids. Trends Cancer 2, 176–190 (2016). 27135056 10.1016/j.trecan.2016.03.004 PMC4847151

[b142] Lancaster M. A. & Knoblich J. A. Organogenesis in a dish: modeling development and disease using organoid technologies. Science 345, 1247125 (2014). 25035496 10.1126/science.1247125

[b143] Papapetrou E. P. Patient-derived induced pluripotent stem cells in cancer research and precision oncology. Nat. Med. 22, 1392–1401 (2016). 27923030 10.1038/nm.4238 PMC5233709

[b144] Lim K. L. . Reprogramming cancer cells: overview & current progress. Expert Opin. Biol. Ther. 16, 941–951 (2016). 27070264 10.1517/14712598.2016.1174211

[b145] Kumano K. . Generation of induced pluripotent stem cells from primary chronic myelogenous leukemia patient samples. Blood 119, 6234–6242 (2012). 22592606 10.1182/blood-2011-07-367441

[b146] Carette J. E. . Generation of iPSCs from cultured human malignant cells. Blood 115, 4039–4042 (2010). 20233975 10.1182/blood-2009-07-231845 PMC2875096

[b147] Hu K. & Slukvin I. in Pluripotent Stem Cells: Methods and Protocols (eds Lakshmipathy U., Vemuri M. C. 163–176Humana Press (2013).

[b148] Kotini A., Dolezal E. K., Nimer S. & Papapetrou E. P. An iPSC-based model Of MDS for phenotype-driven gene and drug discovery. Blood 122, 859–859 (2013).23929837

[b149] Ye Z. . Human-induced pluripotent stem cells from blood cells of healthy donors and patients with acquired blood disorders. Blood 114, 5473–5480 (2009). 19797525 10.1182/blood-2009-04-217406 PMC2798863

[b150] Gandre-Babbe S. . Patient-derived induced pluripotent stem cells recapitulate hematopoietic abnormalities of juvenile myelomonocytic leukemia. Blood 121, 4925–4929 (2013). 23620576 10.1182/blood-2013-01-478412 PMC3682343

[b151] Muñoz-López A. . Development refractoriness of MLL-rearranged human B cell acute leukemias to reprogramming into pluripotency. Stem Cell Rep. 7, 602–618 (2016). 10.1016/j.stemcr.2016.08.013 PMC5063541 27666791

[b152] Kim J. . An iPSC line from human pancreatic ductal adenocarcinoma undergoes early to invasive stages of pancreatic cancer progression. Cell Rep. 3, 2088–2099 (2013). 23791528 10.1016/j.celrep.2013.05.036 PMC3726210

[b153] Hadoux J. . Generation of an induced pluripotent stem cell line from a patient with hereditary multiple endocrine neoplasia 2A (MEN2A) syndrome with RET mutation. Stem Cell Res. 17, 154–157 (2016). 27558615 10.1016/j.scr.2016.06.008

[b154] Iskender B., Izgi K. & Canatan H. Reprogramming bladder cancer cells for studying cancer initiation and progression. Tumour Biol. 37, 13237–13245 (2016). 27456363 10.1007/s13277-016-5226-4

[b155] Lee D. F. . Modeling familial cancer with induced pluripotent stem cells. Cell 161, 240–254 (2015). 25860607 10.1016/j.cell.2015.02.045 PMC4397979

[b156] Ron-Bigger S. . Aberrant epigenetic silencing of tumor suppressor genes is reversed by direct reprogramming. Stem Cells 28, 1349–1354 (2010). 20572015 10.1002/stem.468

[b157] Ohnishi K. . Premature termination of reprogramming *in vivo* leads to cancer development through altered epigenetic regulation. Cell 156, 663–677 (2014). 24529372 10.1016/j.cell.2014.01.005

[b158] Choi P. S. & Meyerson M. Targeted genomic rearrangements using CRISPR/Cas technology. Nat. Commun. 5, 3728 (2014). 24759083 10.1038/ncomms4728 PMC4170920

[b159] Chen C. . MLL3 is a haploinsufficient 7q tumor suppressor in acute myeloid leukemia. Cancer Cell 25, 652–665 (2014). 24794707 10.1016/j.ccr.2014.03.016 PMC4206212

[b160] Torres R. . Engineering human tumour-associated chromosomal translocations with the RNA-guided CRISPR-Cas9 system. Nat. Commun. 5, 3964 (2014). 24888982 10.1038/ncomms4964

[b161] Heckl D. . Generation of mouse models of myeloid malignancy with combinatorial genetic lesions using CRISPR-Cas9 genome editing. Nat. Biotechnol. 32, 941–946 (2014). 24952903 10.1038/nbt.2951 PMC4160386

[b162] Xue W. . CRISPR-mediated direct mutation of cancer genes in the mouse liver. Nature 514, 380–384 (2014). 25119044 10.1038/nature13589 PMC4199937

[b163] Guernet A. . CRISPR-Barcoding for intratumor genetic heterogeneity modeling and functional analysis of oncogenic driver mutations. Mol. Cell 63, 526–538 (2016). 27453044 10.1016/j.molcel.2016.06.017 PMC5537739

[b164] Frieda K. L. . Synthetic recording and in situ readout of lineage information in single cells. Nature 541, 107–111 (2017). 27869821 10.1038/nature20777 PMC6487260

[b165] Qin H., Malek S., Cowell J. K. & Ren M. Transformation of human CD34+hematopoietic progenitor cells with DEK-NUP214 induces AML in an immunocompromised mouse model. Oncogene 35, 5686–5691 (2016). 27065320 10.1038/onc.2016.118 PMC5064821

[b166] Ren M. . Development of ZMYM2-FGFR1 driven AML in human CD34+ cells in immunocompromised mice. Int. J. Cancer 139, 836–840 (2016). 27005999 10.1002/ijc.30100 PMC5754922

